# The impact of targeting repetitive BamHI-W sequences on the sensitivity and precision of EBV DNA quantification

**DOI:** 10.1371/journal.pone.0183856

**Published:** 2017-08-29

**Authors:** Armen Sanosyan, Alexis Fayd’herbe de Maudave, Karine Bollore, Valérie Zimmermann, Vincent Foulongne, Philippe Van de Perre, Edouard Tuaillon

**Affiliations:** 1 CHU Montpellier, Montpellier, France; 2 Pathogenesis and Control of Chronic Infections, INSERM, EFS, Université de Montpellier, Montpellier, France; 3 Institut de Génétique Moléculaire de Montpellier, Centre National de la Recherche Scientifiquem, UMR 5535, Université de Montpellier, Montpellier, France; University of British Columbia, CANADA

## Abstract

**Background:**

Viral load monitoring and early Epstein-Barr virus (EBV) DNA detection are essential in routine laboratory testing, especially in preemptive management of Post-transplant Lymphoproliferative Disorder. Targeting the repetitive BamHI-W sequence was shown to increase the sensitivity of EBV DNA quantification, but the variability of BamHI-W reiterations was suggested to be a source of quantification bias. We aimed to assess the extent of variability associated with BamHI-W PCR and its impact on the sensitivity of EBV DNA quantification using the 1st WHO international standard, EBV strains and clinical samples.

**Methods:**

Repetitive BamHI-W- and LMP2 single- sequences were amplified by in-house qPCRs and BXLF-1 sequence by a commercial assay (EBV R-gene™, BioMerieux). Linearity and limits of detection of in-house methods were assessed. The impact of repeated versus single target sequences on EBV DNA quantification precision was tested on B95.8 and Raji cell lines, possessing 11 and 7 copies of the BamHI-W sequence, respectively, and on clinical samples.

**Results:**

BamHI-W qPCR demonstrated a lower limit of detection compared to LMP2 qPCR (2.33 log_10_ versus 3.08 log_10_ IU/mL; P = 0.0002). BamHI-W qPCR underestimated the EBV DNA load on Raji strain which contained fewer BamHI-W copies than the WHO standard derived from the B95.8 EBV strain (mean bias: - 0.21 log_10_; 95% CI, -0.54 to 0.12). Comparison of BamHI-W qPCR versus LMP2 and BXLF-1 qPCR showed an acceptable variability between EBV DNA levels in clinical samples with the mean bias being within 0.5 log_10_ IU/mL EBV DNA, whereas a better quantitative concordance was observed between LMP2 and BXLF-1 assays.

**Conclusions:**

Targeting BamHI-W resulted to a higher sensitivity compared to LMP2 but the variable reiterations of BamHI-W segment are associated with higher quantification variability. BamHI-W can be considered for clinical and therapeutic monitoring to detect an early EBV DNA and a dynamic change in viral load.

## Introduction

Epstein-Barr virus (EBV) is a member of gamma herpesviruses subfamily which infects more than 95% of adults [1]. Although generally asymptomatic EBV is responsible for Infectious Mononucleosis, an often self-limited disease of adolescents characterized with fever, lymphadenopathy, splenomegaly and atypical lymphocytes [2]. The importance of EBV rose in the modern era of immunosuppression with AIDS, transplants or cancer chemotherapy, where it was demonstrated that high EBV viral load is associated with the risk of lymphoproliferative disorders [3]. EBV DNA measurement in peripheral blood compartments became routine practice to help diagnose, monitor, prevent and treat the post-transplant lymphoproliferative disorder (PTLD) in immunocompromised recipients [4,5]. Although all three blood compartments: whole blood; Peripheral Blood Mononuclear Cells (PBMC) and plasma are appropriate specimens for EBV DNA monitoring, recent evidence suggests plasma to be more indicative for EBV-associated PTLD [5,6].

Sensitive and accurate detection of EBV DNA at the early stages of the PTLD remains crucial in the monitoring of transplanted patients [3,5,7]. Despite the existence of commercial kits, clinical laboratories continue to use in-house real-time PCR (qPCR) assays [4]. Additionally, World Health Organization (WHO) implemented the EBV DNA international standard that was produced from B95.8 EBV strain to standardize the assays and reduce the inter-laboratory variability by using international units (IU) [8]. The introduction of the “1st WHO International standard for Epstein—Barr virus for Nucleic Acid Amplification Technique” (NIBSC code: 09/260) aimed to provide the laboratories with a single reference source of defined EBV DNA [8].

**Fig 1:**
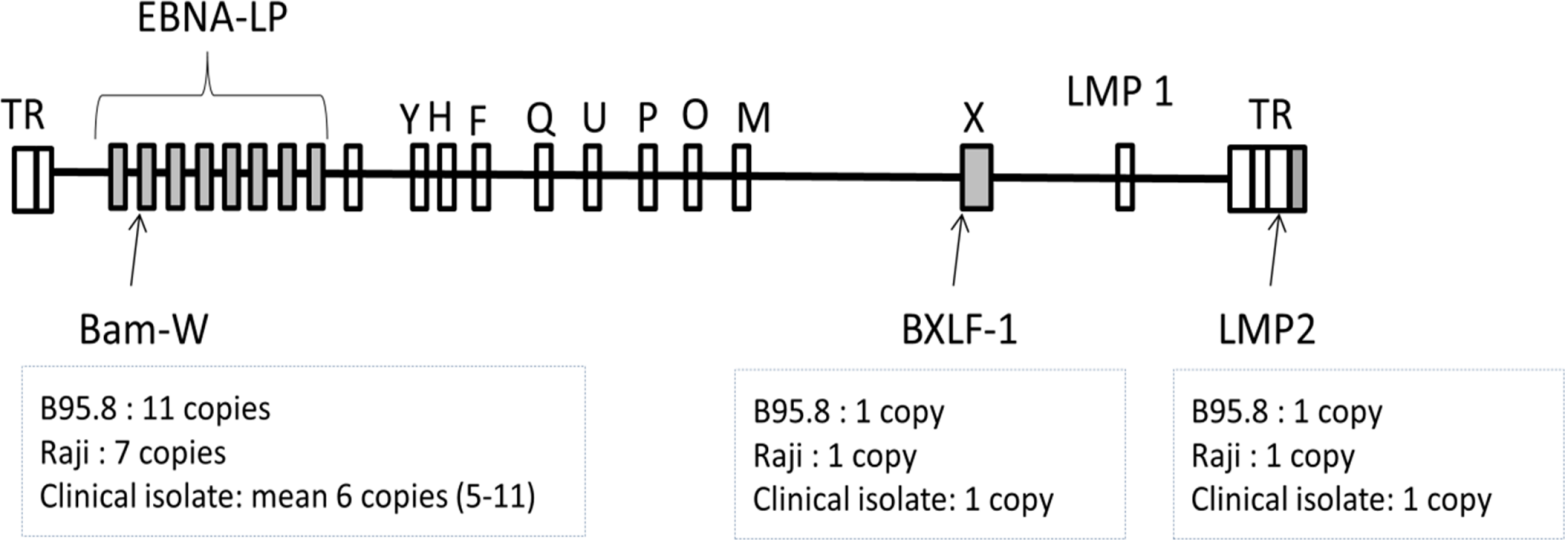
Schematic representation of linear form of EBV genome mapping the Bam-W reiterations and other targets of our study (shaded areas). TR–terminal repeats; LMP–latent membrane protein; letters represent BamHI degraded fragments of EBV genome.

One possible mean to increase the sensitivity of the qPCR assay is to target the repetitive sequences of the pathogen genome [9,10]. The assays amplifying BamHI-W (Bam-W) fragment, which is a tandem reiterated sequence found in the EBNA-LP (EBV nuclear antigen leader protein) region of EBV genome were shown to be more sensitive compared to a single sequence qPCRs [11,12]. Bam-W represents a highly conserved sequence that is reiterated in average 6 times per EBV isolate [13,14]. However, the reiteration of Bam-W region is inconstant, ranging from 5 to 11 copies in different isolates (Fig 1) [14,15]. Variability in Bam-W copy numbers may add an imprecision in EBV quantification due to the differences between the Bam-W sequence numbers in the quantification standards and tested samples [11]. Although the precision of the assay was questioned, studies continue to demonstrate the effectiveness and good quantitative agreement of Bam-W qPCR [16,17]. However, to the best of our knowledge no study has explored the extent of EBV DNA quantification imprecisions and the gain of the sensitivity determined by the variability of Bam-W reiterations.

In the current study we developed two in-house qPCR assays targeting the single and repetitive sequences of EBV genome and compared the results between in-house and commercial tests. The impact of targeting repetitive Bam-W sequence on the sensitivity and precision of EBV DNA quantification was evaluated across the wide range of EBV viral loads using EBV strains and clinical samples.

## Material and methods

### Sample preparation and quantitative standards

EBV strains from B95.8 and Raji cell lines (kindly donated by Prof. Claude Desgranges), were used in this study. B95.8 (GenBank: V01555.2) and Raji (GenBank: KF717093.1) complete genome sequences contain 11 and 7 Bam-W copy numbers, respectively. Supernatants from Raji and B95.8 EBV cell lines were harvested after three days of incubation without any stimulation, tested by the EBV R-gene™ kit (Biomerieux, Marcy l'Etoile, France) and serially diluted from 2.6 to 6.7 log_10_ IU/mL. Thirty six clinical samples of whole blood collected from patients visiting University Hospital Center of Montpellier were also included with 25 positives and 11 negatives for EBV DNA. All participants had provided verbal informed consent at the initial workup, and the University Hospital Center of Montpellier ethic committee approved the collection with the reference: DC-2015-2473 for this study (Comité de Protection des Personnes Sud Méditerranée III). The 1st WHO international standard for EBV was ordered from the National Institute for Biological Standards and Control (NIBSC, UK) and dilutions in EBV DNA-negative plasma from blood donor were used for limit of detection (LOD) assays and serial 10-fold dilutions ranging from 5 000 000 IU/mL (6.70 log_10_ IU/mL) to 500 IU/mL (2.70 log_10_ IU/mL) were used for estimation of the linear dynamic ranges of in-house tests.

### DNA extraction

DNA from 200 μl of whole blood was extracted and eluted into a final volume of 60 μl using a QIAsymphony DNA Mini Kit on the QIAsymphony SP automated DNA extractor (Qiagen, Hilden, Germany).

EBV DNA from cell supernatants and 1st WHO International standard was extracted from 200 μl samples, eluted in 50 μl using the QIAamp DNA mini Kit (Qiagen) on the QIAcube automated DNA extractor (Qiagen).

### EBV DNA quantification

Assays were performed using a LightCycler 480 instrument (Roche, Mannheim, Germany) in 96-well plate.

Three different targets were used to quantify EBV DNA in the study samples: a repetitive highly conserved Bam-W and a single LMP2 sequences for in-house qPCRs; and a BXLF-1 gene for the commercial EBV R-gene kit.

In-house qPCR reactions were performed in 20 μl final volume containing 5 μl of DNA and 5x DNA polymerase mix (Omunis, Clapiers, France) with the following thermocycling conditions: activation—95°C for 12 minutes and amplification—95°C for 15 seconds following 60°C for 1 minute during 50 cycles. The sequences and concentrations of the primers and probes were:

**Bam-W** qPCR [18]– 400 nM of forward (AGTGGGCTTGTTTGTGACTTCA) and reverse (GGACTCCTGGCGCTCTGAT) primers and 125 nM of TaqMan probe (6FAM-TTACGTAAGCCAGACAGCAGCCAATTGTC-TAMRA).

**LMP2** [19]– 600 nM of forward (AGCTGTAACTGTGGTTTCCATGA) and reverse (GCCCCCTGGCGAARAG) primers and 400 nM of probe (6FAM-CTGCTGCTACTGGCTTTCGTCCTCTGG-TAMRA).

Potential cross-reactivity with other viruses or genomic DNA was evaluated by BLAST sequence analysis.

The R-gene PCR targeting the BXLF-1 region of EBV genome was used according to the manufacturer’s recommendations. Analytical performances of R-gene EBV™ are: detection limit of 182 copies/mL on Namalwa cell dilutions (performed by the manufacturer); 500 copies/mL on a whole blood and the linearity ranging from 2.5*10^3^ to 2.5*10^6^ copies/mL [20].

Quantification results for experimental samples were extrapolated based on the standard curve. All viral loads were expressed as IU/mL. All in-house assays and R-gene assay on cell lines were quantified based on calibration curves constructed by WHO EBV standard. EBV DNA loads of clinical samples were converted from copies/mL to IU/mL using the 0.44 conversion factor previously calculated in the clinical laboratory of virology of our institution regarding the workflow used for the EBV viral load determination by a method described elsewhere [21].

### PCR performances and statistics

The linear dynamic ranges of the in-house assays were evaluated by plotting the results of 10-fold serial dilutions of the WHO standard ranging between 5*10^2^ to 5*10^6^ IU/mL in three different runs with 8–13 replicates of each concentration. The linear regression analysis between assigned and observed values was used for linearity assessment. The median values, interquartile ranges of each concentration and regression coefficient were determined.

The capacity to amplify the lower concentrations of EBV DNA was explored by testing the LOD, which assumes the lowest EBV DNA load detected in 95% of the times. To determine the LOD, serial dilutions ranging from 80 to 1000 IU/mL were tested from 4 to 62 times in three different runs by each in-house assay. The loads that were estimated to have 95% of positivity were tested at least 8 times per run, whereas the loads having absolute positivity rate were tested at least 4 times in total. Lower LOD was expected using the Bam-W qPCR. The probit regression analysis with a value equal to 6.65 probability of units was used to determine the LOD for both in-house PCRs as described elsewhere [22]. Values having absolute positivity rate (100% tested positive) do not participate in probit analysis.

Bland-Altman bias plots were used to assess the differences between repeated (Bam-W) and single target qPCR assays (LMP2 and R-gene) on EBV strains and clinical samples. For each plot, mean bias and 95% confidence interval (95% CI) of the bias were calculated and the mean biases were compared using Student’s t-test. EBV strains containing different numbers of Bam-W sequences were used to evaluate the bias in EBV quantification due to a known variation in reiteration of a repeated sequence. Confidence interval of bias was used to test the concordance between results from clinical samples using repeated- versus single-target qPCRs. Wider variations around the mean bias line would reflect variations in the ratio of Bam-W/single target (LMP2 or BXLF-1) in clinical samples. The agreement between the different qPCRs was analyzed using Cohen’s kappa test.

All statistical analyses were done with MS Excel and Graphpad Prism 6.0 (GraphPad Software, Inc., San Diego, CA). Graphpad Quickcalcs online software was used for Student’s t-test and kappa agreement.

## Results

### Analytical performances of Bam-W and LMP2 in-house qPCR assays

**Fig 2:**
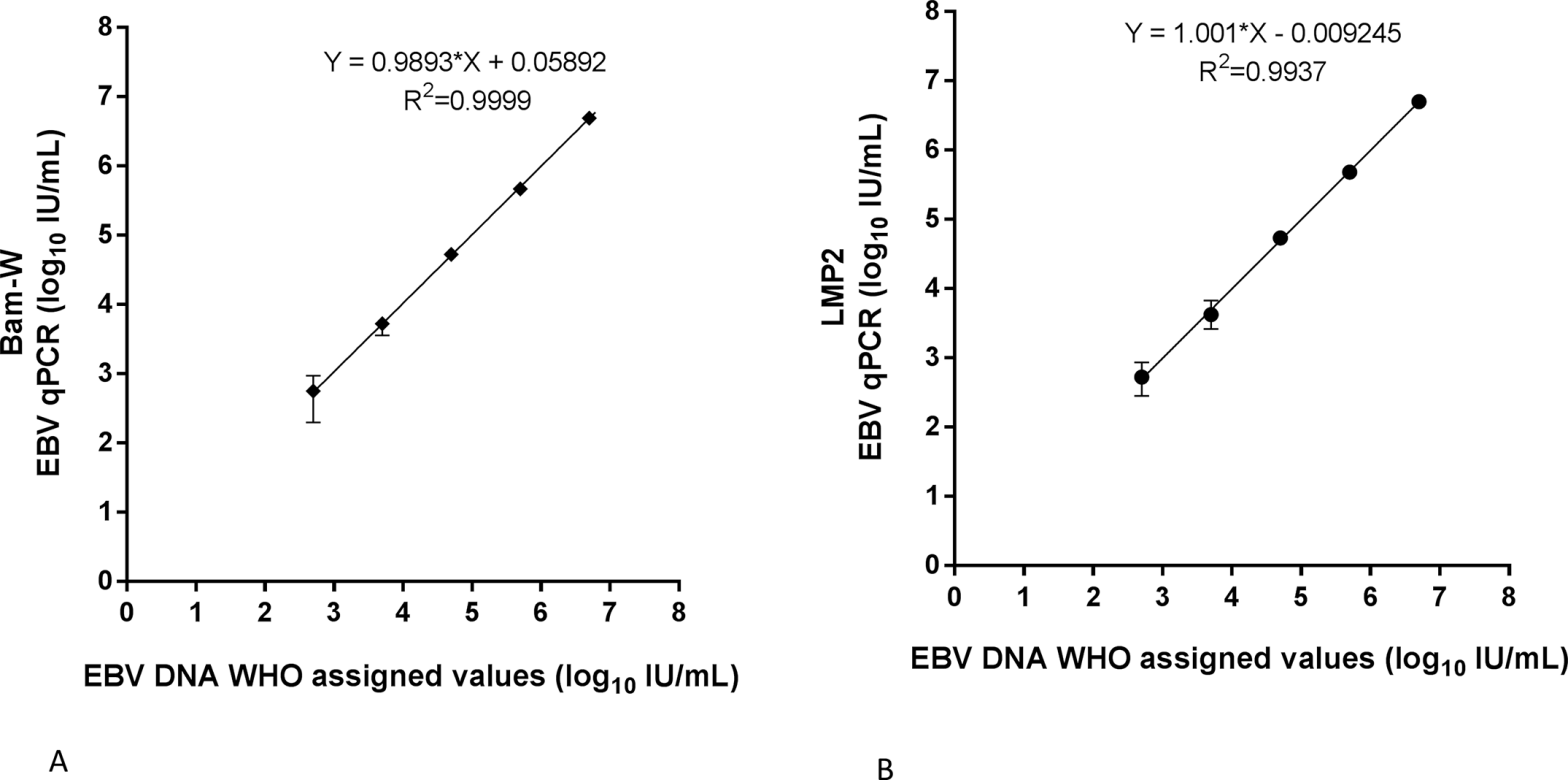
Linearity experiments evaluating reportable ranges of Bam-W repeated- (A) and LMP2 single- (B) sequence qPCRs from 5*10^2^ to 5*10^6^ IU/ml EBV DNA in serial dilutions of 1st WHO international standard. Each point was tested from 8 to 13 replicates. Standard deviation and coefficient of variation of each dilution is presented in the supporting S1 Table.

The Bam-W and LMP2 assays were linear between 500–500 000 IU/mL with regression coefficients of 0.99 (95% CI, 0.97–1.01) and 1.00 (95% CI, 0.98–1.02), respectively. The coefficient of determination (R^2^) was 1.00 for Bam-W assay and 0.99 for LMP2 assay. Inter-quartile ranges were increasingly smaller from low to high concentrations in both tests, S1 Table (Fig 2).

**Fig 3:**
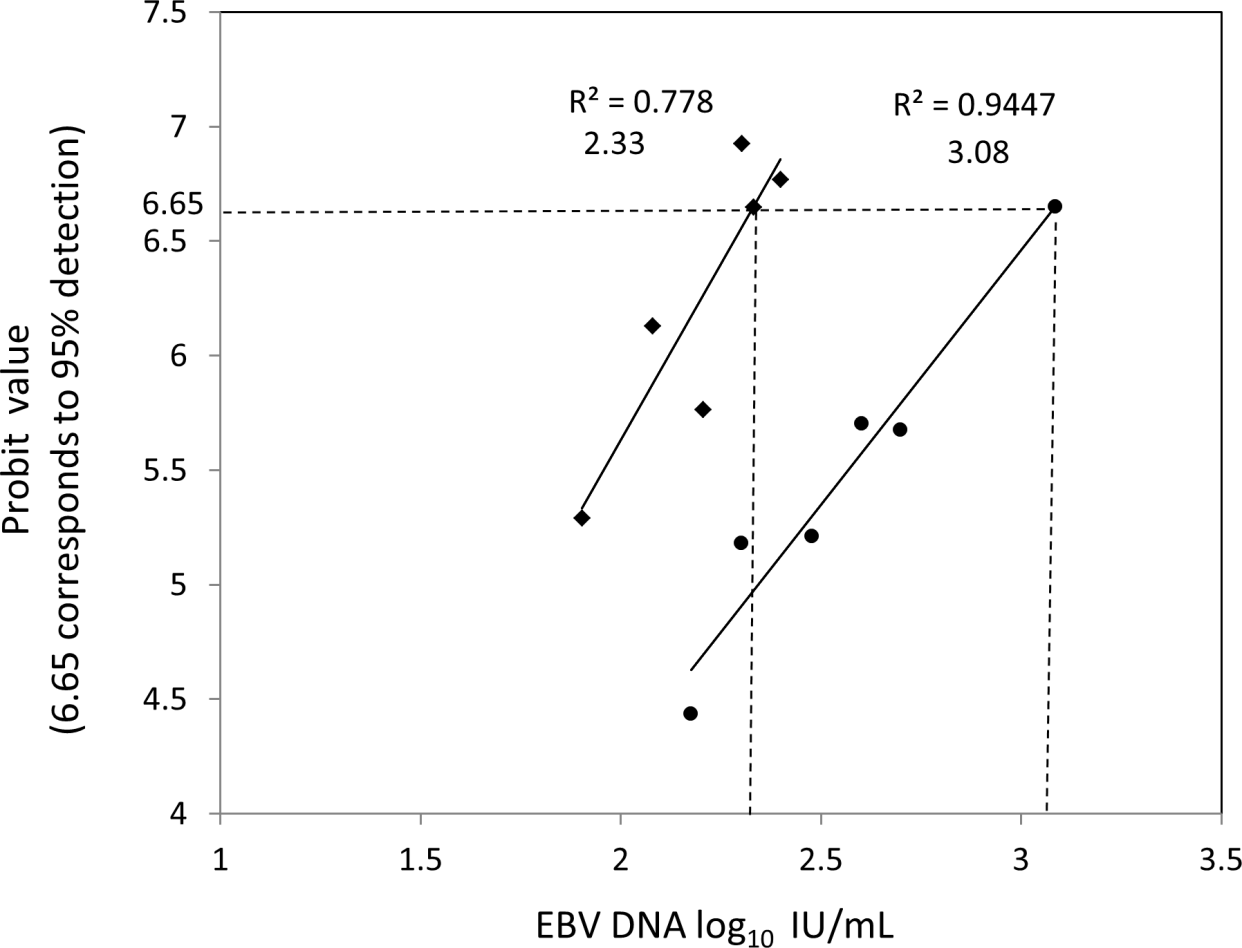
Probit regression analysis determining the limit of detection (the lowest viral load detectable in 95% of the time, corresponding to probit value 6.65) of Bam-W repeated- and LMP2 single- sequence qPCRs. A series of 7 and 6 EBV concentrations for Bam-W and LMP2 qPCRs, respectively, were prepared by spiking 1st WHO international standard in EBV DNA negative plasma. Each dilution was tested in 4 to 62 replicates. The number of replicates, positivity rate and corresponding probit value of each dilution is presented in supporting S2 Table.

A series of dilutions ranging from 1.9 to 3.0 log_10_ (80 to 1000) IU/mL were prepared by spiking the 1st WHO international standard in EBV DNA-negative plasma to evaluate the LOD of Bam-W and LMP2 qPCRs, S2 Table. LOD for Bam-W was set at 214 IU/mL (2.33 log_10_ IU/mL) and for LMP2 at 1215 IU/mL (3.08 log_10_ IU/mL); P = 0.0002 (Fig 3).

### Impact of repeated versus single sequence PCR on EBV DNA quantification on B95.8 and Raji strains

The supernatants of B95.8 and Raji cell lines were tested with Bam-W and LMP2 in-house qPCRs and R-gene kit. Results were compared using Bland-Altman bias plots Table 1.

**Table 1:** Biases, standard deviations and comparison of biases (P values) of Bland-Altman curves on different EBV strains.

	B95.8 (Bias/SD)	Raji (Bias/SD)	P values
LMP 2—R-gene	0.07/0.09	0.15/0.10	0.057
Bam-W—R-gene	0.15/0.18	-0.05/0.09	0.0049
Bam-W—LMP2	0.09/0.12	-0.21/0.17	0.0002

SD–standard deviation

**Fig 4:**
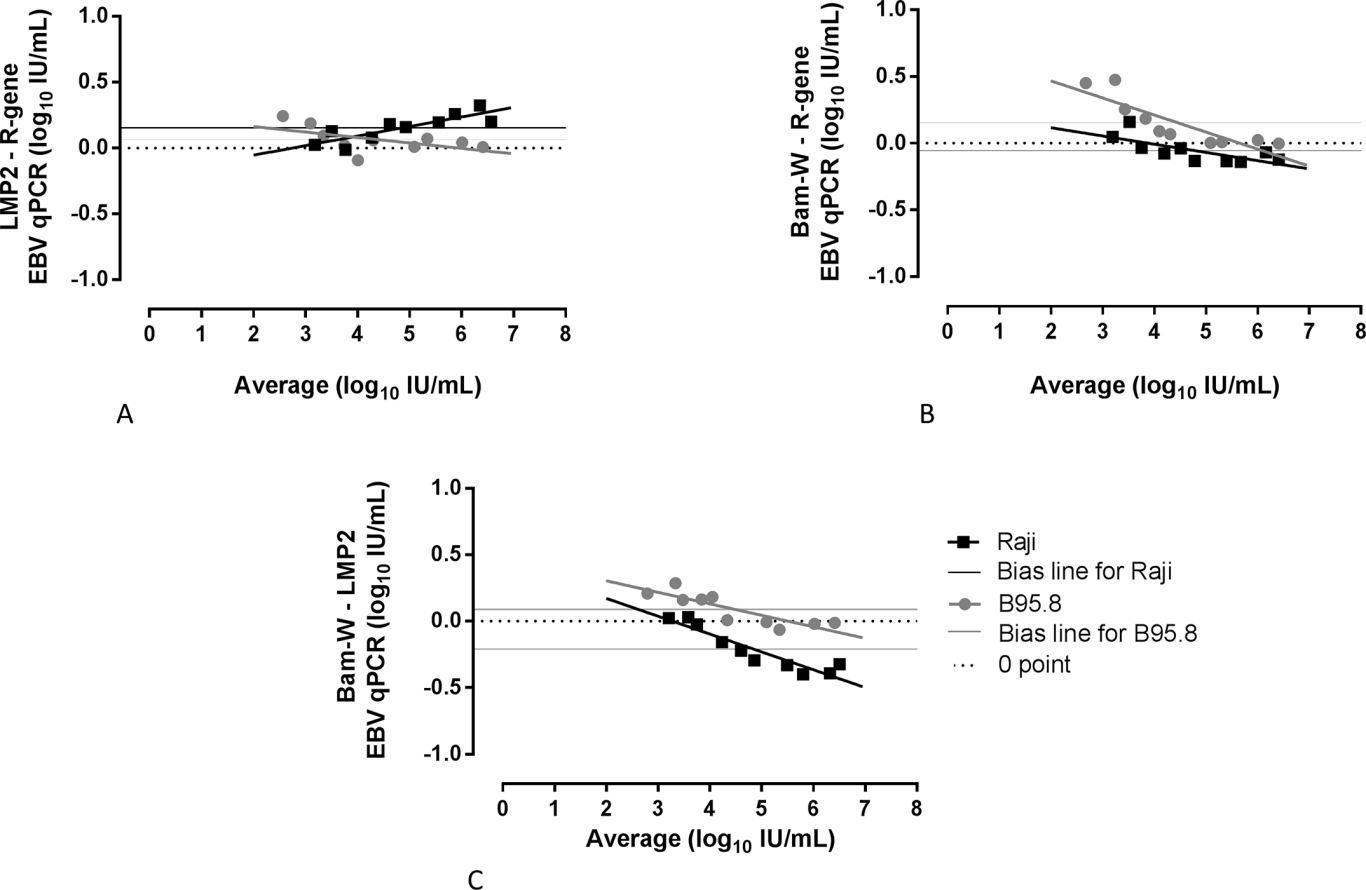
Bland-Altman bias plots for three different quantitative EBV DNA real-time PCR assays. Ten serial dilutions of Raji and B95.8 cell line supernatants were tested for EBV DNA quantification by LMP2 and EBV R-gene qPCRs (A). The same samples were tested by Bam-W qPCR compared with R-gene (B) and LMP2 qPCRs (C).

The difference in EBV DNA levels between LMP2 and R-gene assays were 0.15 log_10_ IU/mL (95% CI, -0.05 to 0.36) for Raji, and 0.07 log_10_ IU/mL (95% CI, -0.12 to 0.25) for B95.8 strain. The biases in EBV DNA in between the two cell lines were non-significant (P = 0.057) (Fig 4A). The differences between EBV DNA levels using in-house repeated target Bam-W qPCR versus R-gene qPCR were lower on Raji strain compared to B95.8 strain. The biases demonstrated on Bland-Altman plots showed higher EBV DNA levels on B95.8 strain using Bam-W qPCR compared to R-gene (bias, 0.15 log_10_ IU/mL; 95% CI,-0.20 to 0.51); whereas a negative bias was observed on Raji strain (bias, -0.05 log_10_ IU/mL; 95% CI, -0.24 to 0.13, P = 0.0049) (Fig 4B). Higher levels of discrepancies were observed in the lower concentrations of EBV DNA. Comparison of Bam-W versus LMP2 (Fig 4C) demonstrated a clear separation between the EBV DNA of the two strains with biases equal to -0.21 log_10_ IU/mL (95% CI, -0.54 to 0.12) on Raji strain and 0.09 log_10_ IU/mL (95% CI, -0.15 to 0.33) on B95.8 strain (P = 0.0002).

### Consequences of targeting repeated sequence on commutability of qPCR results

**Fig 5:**
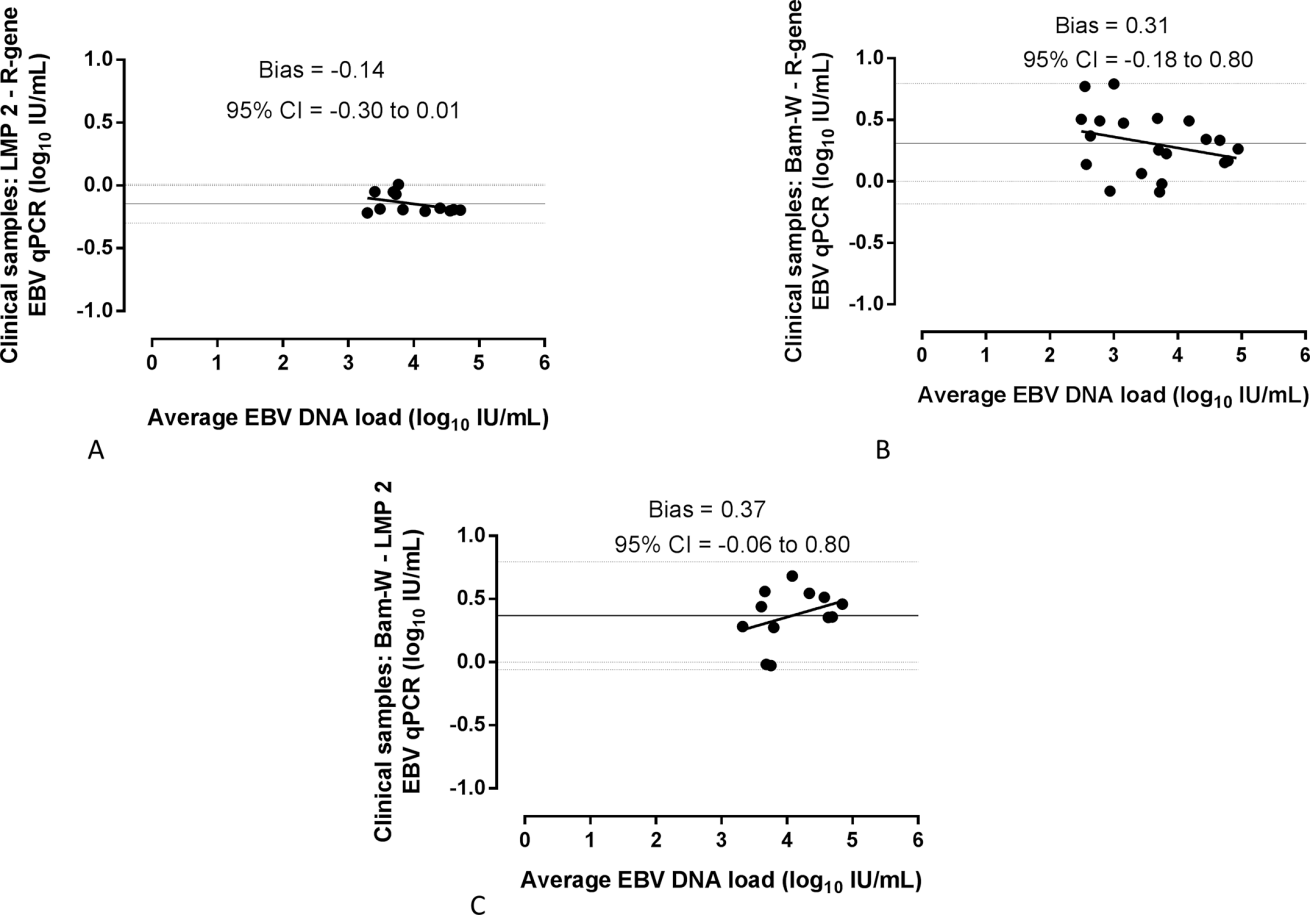
Bland-Altman bias plots comparing Bam-W repeated- and LMP2 single- sequence qPCRs on whole blood extracts of clinical samples. Values having EBV DNA load equal or higher than LOD of LMP2 (A) and Bam-W (B) in-house qPCR assays were compared with the initial clinical values (equal or higher than LOD) performed by EBV R-gene commercial kit, and with each other (C). Dotted lines represent the borders of 95% CI and 0 point.

Testing whole blood extracts from clinical samples a good agreement of EBV DNA detection between commercial R-gene kit and in-house qPCRs was observed with following kappa values: 0.87 (95% CI, 0.69–1.00) for Bam-W and 0.66 (95% CI, 0.43–0.89) for LMP2 Table 2. EBV DNA load dispersion due to inconsistent copies of Bam-W was observed on the plots comparing Bam-W qPCR to LMP2 or R-gene qPCRs in clinical isolates (Fig 5). The distance between the bias confidence interval bands was from -0.30 to 0.01 for LMP2 versus R-gene (Fig 5A); from -0.18 to 0.80 log_10_ IU/mL for Bam-W versus R-gene (Fig 5B) and from -0.05 to 0.80 for Bam-W versus LMP2 (Fig 5C). However, all biases are within the acceptable ± 0.5 log_10_ IU/mL range of difference.

**Table 2:** Cohen’s kappa agreements of EBV DNA between qualitative results obtained from whole blood clinical sample of R-gene commercial kit and laboratory in-house Bam-W and LMP2 qPCR assays.

		R-gene positive samples (n = 25)	R-gene negative samples (n = 11)	Kappa agreement (95% CI)
Bam-W	positive	24	1	0.87 (0.69–1.00)
	negative	1	10	
LMP2	positive	19	0	0.66 (0.43–0.89)
	negative	6	11	

## Discussion

This study highlighted the impact of targeting Bam-W repetitive sequences on the sensitivity and quantification precision of EBV viral load. Targeting Bam-W region was shown to increase the sensitivity but the variations in the reiteration number has long been suggested as a potential drawback especially in clinical settings [11,23].

The probit regression analyses on the plasma dilutions of WHO standard proved that the repeated Bam-W sequence qPCR had a LOD lower than LMP2 single repeat qPCR. Compared to LMP2, the gain of LOD targeting Bam-W was over 0.5 log_10_ IU/mL, which can be considered significant for qPCR method [21]. Based on the characteristics provided by the manufacturer, the LOD of the Bam-W PCR and R-gene EBV kit appeared comparable. In a study evaluating EBV PCR for diagnosing and monitoring PTLD Tsai et al. compared EBV plasma loads measured with 3 different qPCR methods (EBNA-1, LMP2 and EBER) and have shown that EBNA-1 had higher negative predictive value due to its higher sensitivity [24]. Further studies remain needed to evaluate the possible benefit of detection of low level of EBV DNA in plasma to predict disease progression or resolution of PTLD under therapy.

The bias in EBV DNA quantification related to variation in Bam-W repeat number was explored on Raji versus B95.8 strain. Theoretical underestimation on the Raji strain having 7 copies relative to the B95.8 strain having 11 copies can be calculated by logarithmic subtraction of the number of copies from the two cell lines (log_10_7—log_10_11 = -0.196 log_10_ IU/mL) which was close to the observed bias (-0.21 log_10_ IU/mL).

WHO standard made from B95.8 cell line contains 11 reiterations of Bam-W sequences and determines a possible quantification disagreement between the tested samples with variable Bam-W [16,25]. Our results confirmed this assumption, since the plots comparing the results of Bam-W qPCR with LMP2 or R-gene qPCRs in clinical samples made evident a visible dispersion of EBV DNA around the bias lines, whereas a narrower variation of bias confidence interval was observed on LMP2 versus R-gene plot. Abeynayake et al. have demonstrated commutable performances between in-house Bam-W qPCR and Artus (EBNA-1) commercial tests in clinical samples using WHO EBV standard. An acceptable difference of 0.5 log_10_ UI/mL was detected for around 90% of cases showing a good agreement between the two tests [16]. Testing serial dilutions (2.6–6.7 log_10_ IU/mL) of different cell lines we also obtained a difference between repetitive- and single- sequence qPCRs close to theoretic calculation that is within the acceptable 0.5 log_10_ IU/mL range [21]. In a study using plasmids containing 1 copy of each target for standard curve construction Le et al. compared three different qPCR assays (Bam-W; LMP2 and Polymerase 1). They demonstrated that although Bam-W overestimated the viral load in cell lines, in clinical samples all three can be interchangeably used for monitoring EBV DNA in nasopharyngeal carcinoma, however none of the authors described the limit of detection and sensitivity gain of the assays [15].

This suboptimal comparability of qPCR results may affect the interpretation when threshold is applied. The management of PTLD still lacks a common consensus around the EBV DNA threshold for preemptive therapy. Three EBV DNA thresholds of 1000, 10 000, 40 000 copies/mL have been suggested for Rituximab therapy [26–28].

Using these thresholds in our clinical samples, we have detected 7 disagreements between Bam-W and R-gene tests and 2 between LMP2 and R-gene test. Hence, the repeated sequence qPCR yielded to higher sensitivity but also to higher variability oscillating around the bias line. Molecular assays targeting single sequence should be preferred for PTLD therapeutic management if a strict threshold is applied for EBV DNA monitoring. Although the introduction of WHO standard harmonized the assays by providing a common calibrator and reporting units, the lack of commonly accepted viral load threshold and specimen type for diagnosing EBV-related diseases still remain a substantial source of difficulties for interpretation of the results and for comparisons across laboratories.

In perspective, the interest of Bam-W qPCR may not only be confined in EBV DNA load monitoring but may also represent an additional source of information in the monitoring of EBV associated diseases. Ishii et al. demonstrated a significant correlation between Bam-W and LMP1 plasma qPCRs. However EBV DNA quantified by two methods did not exhibit the same dynamic change during the treatment of NK/T cell tumors [29].

EBNA-LP protein substantially contributes to EBV infected B cell oncogenic transformation [30,31]. The Bam-W reiteration number in EBNA-LP gene determines the size and thus the functional ability of EBNA-LP [12]. By using recombinant EBV, Tierney et al. have shown that the optimal transformation capacity of EBNA LP is with 5 to 8 Bam-W repeats, while EBV virions with W reiterations under 5 copies significantly decreased their transformation ability [12].

In conclusion, in-house Bam-W repeated target qPCR is more sensitive than single repeat LMP2 qPCR with significantly lower limit of detection. Despite the quantification imprecisions imposed by the variable numbers of Bam-W reiteration an acceptable agreement exists between Bam-W and single target qPCR assays. Hence, Bam-W can be considered in further studies for the early detection of EBV DNA and for therapeutic monitoring based on EBV viral load dynamic changes.

## Supporting information

S1 Table Reportable range of two in-house EBV DNA quantification assays on serial dilutions of the 1st WHO international standard for EBV. (DOCX)

S2 Table Limit of detection using probit regression analysis. (DOCX)

S1 File Raw data PLOS ONE data 19.05.2017. (XLSX)
