# Supplementary material for: The impact of targeting repetitive BamHI-W sequences on the sensitivity and precision of EBV DNA quantification
Source: PLoS One. 2017 Aug 29;12(8):e0183856. doi: 10.1371/journal.pone.0183856 (PMC5574619; doi:10.1371/journal.pone.0183856)
Supplement: S1 Table — (DOCX) [file pone.0183856.s001.docx]

**Supporting Table S1. Reportable range of two in-house EBV DNA quantification assays on serial dilutions of the 1st WHO international standard for EBV**

|  | Serial dilutions of the 1st WHO standard | Assigned values  (IU/ml) | Assigned values  (log_10_ IU/ml) | qPCR mean  (log_10_ IU/ml) | SD | CV (%) | Number of experiments | Total number of replicates |
| --- | --- | --- | --- | --- | --- | --- | --- | --- |
| Bam-W  qPCR | Std 1 | 5*10^6^ | 6.70 | 6.69 | 0.03 | 0.49 | 3 | 8 |
|  | Std 2 | 5*10^5^ | 5.70 | 5.68 | 0.03 | 0.44 | 3 | 13 |
|  | Std 3 | 5*10^4^ | 4.70 | 4.73 | 0.03 | 0.66 | 3 | 13 |
|  | Std 4 | 5*10^3^ | 3.70 | 3.71 | 0.07 | 1.81 | 3 | 13 |
|  | Std 5 | 5*10^2^ | 2.70 | 2.73 | 0.23 | 8.36 | 3 | 13 |
| LMP2  qPCR | Std 1 | 5*10^6^ | 6.70 | 6.70 | 0.01 | 0.15 | 3 | 8 |
|  | Std 2 | 5*10^5^ | 5.70 | 5.68 | 0.02 | 0.41 | 3 | 13 |
|  | Std 3 | 5*10^4^ | 4.70 | 4.73 | 0.03 | 0.71 | 3 | 13 |
|  | Std 4 | 5*10^3^ | 3.70 | 3.63 | 0.14 | 3.76 | 3 | 13 |
|  | Std 5 | 5*10^2^ | 2.70 | 2.72 | 0.17 | 6.18 | 3 | 13 |

Std - standard; SD - standard deviation; CV - coefficient of variation
