# Supplementary material for: The impact of targeting repetitive BamHI-W sequences on the sensitivity and precision of EBV DNA quantification
Source: PLoS One. 2017 Aug 29;12(8):e0183856. doi: 10.1371/journal.pone.0183856 (PMC5574619; doi:10.1371/journal.pone.0183856)
Supplement: S2 Table — (DOCX) [file pone.0183856.s002.docx]

**Supporting Table S2. Limit of detection using probit regression analysis.**

|  | IU/ml | Log_10_ IU/ml | Replicates | Positives | Positivity rate | Probit value |
| --- | --- | --- | --- | --- | --- | --- |
| Bam-W qPCR | 500 | 2.70 | 4 | 4 | 1.00 | N/A |
|  | 400 | 2.60 | 4 | 4 | 1.00 | N/A |
|  | 250 | 2.40 | 26 | 25 | 0.96 | 6.77 |
|  | 200 | 2.30 | 37 | 36 | 0.97 | 6.93 |
|  | 160 | 2.20 | 36 | 28 | 0.78 | 5.76 |
|  | 120 | 2.08 | 62 | 54 | 0.87 | 6.13 |
|  | 80 | 1.90 | 26 | 16 | 0.62 | 5.29 |
| LMP2  qPCR | 1000 | 3.00 | 21 | 21 | 1.00 | N/A |
|  | 500 | 2.70 | 28 | 21 | 0.75 | 5.67 |
|  | 400 | 2.60 | 29 | 22 | 0.76 | 5.70 |
|  | 300 | 2.48 | 24 | 14 | 0.58 | 5.21 |
|  | 200 | 2.30 | 14 | 8 | 0.57 | 5.18 |
|  | 150 | 2.18 | 14 | 4 | 0.29 | 4.43 |

N/A - not applicable
